# Serum Protein *N*-Glycosylation Changes with Rheumatoid Arthritis Disease Activity during and after Pregnancy

**DOI:** 10.3389/fmed.2017.00241

**Published:** 2018-01-08

**Authors:** Karli R. Reiding, Gerda C. M. Vreeker, Albert Bondt, Marco R. Bladergroen, Johanna M. W. Hazes, Yuri E. M. van der Burgt, Manfred Wuhrer, Radboud J. E. M. Dolhain

**Affiliations:** ^1^Center for Proteomics and Metabolomics, Leiden University Medical Center, Leiden, Netherlands; ^2^Department of Rheumatology, Leiden University Medical Center, Leiden, Netherlands; ^3^Department of Rheumatology, Erasmus Medical Center, Rotterdam, Netherlands; ^4^Department of Clinical Chemistry, Leiden University Medical Center, Leiden, Netherlands

**Keywords:** rheumatoid arthritis, pregnancy, disease activity score 28 C-reactive protein, glycosylation, total serum protein *N*-glycosylation, ethyl esterification matrix-assisted laser desorption/ionization time-of-flight mass spectrometry

## Abstract

Symptoms of rheumatoid arthritis (RA) improve during pregnancy, a phenomenon that was found to be associated with *N*-glycosylation changes of immunoglobulin G. Recent advances in high-throughput glycosylation analysis allow the assessment of the *N*-glycome of human sera as well. The aim of this study was to identify new protein *N*-glycosylation properties that associate with changes in RA disease activity during and after pregnancy. A longitudinal cohort of serum samples was collected during 285 pregnancies (32 control individuals and 253 RA patients). Per individual one sample was collected before conception, three during pregnancy, and three after delivery. Released serum protein *N*-glycans were measured by matrix-assisted laser desorption/ionization time-of-flight mass spectrometry (MALDI-TOF-MS) after employing chemical modification of the sialic acids to allow discrimination of sialic acid linkage isomers. Serum protein *N*-glycosylation showed strongly modified during pregnancy, with similar changes visible in control individuals and RA pregnancies. Namely, a decrease in bisection and an increase in galactosylation in diantennary glycans were found, as well as an increase in tri- and tetraantennary species and α2,3-linked sialylation thereof. The change in RA disease activity [DAS28(3)-CRP] proved negatively associated with the galactosylation of diantennary *N*-glycans, and positively with the sialylation of triantennary fucosylated species (A3FGS). While the protein source of the novel finding A3FGS is thus far unknown, its further study may improve our understanding of the etiology of RA disease severity.

## Introduction

Rheumatoid arthritis (RA) is a prevalent autoimmune disease affecting up to 1% of the adult population in developed regions (1, 2). Its main characteristic is a symmetrical polyarthritis involving predominantly the hand and foot joints, although every organ system may be involved. The disease is more frequently diagnosed in women than in men and is more common with increasing age (1, 3). The potential causes of RA are diverse in nature (4, 5), but a role for T-cells, antibody-producing B-cells, but also monocytes/macrophages has been suggested (1, 6). Interestingly, in many female RA patients, an improvement in RA disease severity is reported during pregnancy, as well as a relapse thereof after delivery (7–10). The reasons for these changes in disease activity are still poorly understood, but are of importance to understand the etiology of RA, and may possibly provide leads for new modes of (personalized) treatment.

**Figure 1:**
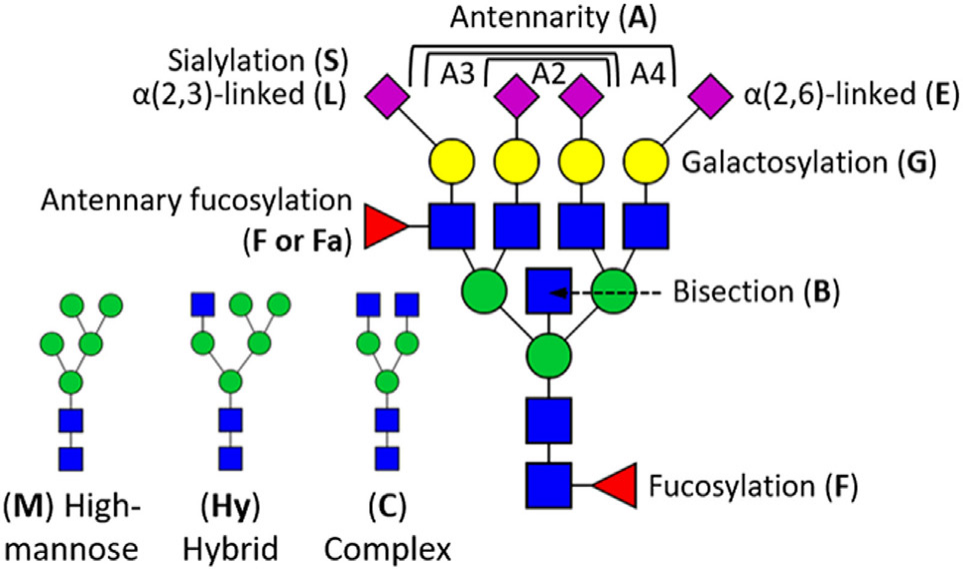
Schematic overview of glycosylation traits derived from human serum proteins. *N*-Glycan structures are generalized into high-mannose type (M), hybrid type (Hy), and complex type (C) by the number of mannoses (green circle) and antennary *N*-acetylglucosamines (blue square). High-mannose-type *N*-glycans can have up to nine mannoses, whereas each antennary *N*-acetylglucosamine can be terminally substituted with a galactose (yellow circle), and sialic acid (magenta diamond). Sialic acids (S) can either be α2,3-linked (L) or α2,6-linked (E). *N*-Glycans can further be modified with a fucose (F), optionally at an antennary *N*-acetylglucosamine (Fa), and structures may as well be bisected (B). In case of derived traits, the subject of the calculation is represented by the last letter, e.g., sialylation (S), and the group on which it is calculated by the preceding letters, e.g., triantennary fucosylated species (A3F). This, for instance, translates A3FGS into the sialylation per galactose within triantennary fucosylated species.

Glycosylation, the process of co- and posttranslational protein modification with complex carbohydrates, plays an important role in the interaction, function, and solubility of proteins (11–13). It is expected that more than half of all proteins is glycosylated with one or more *N*-glycans (14), and commonly observed glycoforms range from high-mannose- to complex type with two to four antennae (branching *N*-acetylglucosamines) (Figure 1) (12, 15). These structures may be extended by additional monosaccharides such as a bisecting *N*-acetylglucosamine, as well as galactoses, *N*-acetylneuraminic acids (sialic acids), and fucoses in a variety of different positions and linkages. This leads to a large *N*-glycan diversity, and may also lead to the formation of specific epitopes such as sialyl-Lewis X, which can be recognized by E-selectin (12, 16).

Various proteins have already been found to display altered glycosylation with RA and its disease activity. For instance, the *N*-glycosylation of the fragment-crystallizable (Fc) portion of immunoglobulin G (IgG) shows to differ in galactosylation, bisection, and fucosylation (17–23), and the acute-phase protein alpha-1-acid glycoprotein (also known as orosomucoid) shows differences in antennarity (i.e., the ratio between di-, tri-, and tetraantennary glycans) and fucosylation with RA as well as changes throughout pregnancy (24, 25). Although such an analysis of single proteins is highly informative, additional insights may be gained by a systemic glycomics approach that covers a broad range of protein-linked glycan modifications.

The total serum *N*-glycome (TSNG) comprises the *N*-glycans from all serum proteins, which are to a large extent liver- (acute-phase proteins) and plasma cell-derived (antibodies) (15, 26). Interestingly, the TSNG has been shown, in a small sample set, to differ between healthy individuals and those with RA, and undergoes clear alteration throughout pregnancy and the following postpartum period (27, 28). However, it is hitherto unknown which TSNG characteristics are associated with the disease activity of RA, and changes thereof throughout pregnancy. Recent developments in mass spectrometry (MS)-based high-throughput glycosylation analysis have provided the opportunity to acquire information on TSNG *N*-glycan complexity, antennarity, galactosylation, fucosylation, as well as on the presence and linkage of sialic acids (α2,6- vs. α2,3-linkage) (29, 30). The latter appears to be of high immunological relevance, since the α2,3-linked sialic acids are required for sialyl-Lewis X formation implicated in the interaction with selectins (29, 30).

The objective of the work presented here is to assess the differences in serum *N*-glycosylation throughout pregnancy and the postpartum period in RA patients and to identify the glycosylation properties associated with the changes in disease activity [DAS28(3)-CRP] during pregnancy. To achieve this, we studied the *N*-glycosylation of sera from 253 RA and 32 control pregnancies at seven time points before, during, and after pregnancy by matrix-assisted laser desorption/ionization time-of-flight (MALDI-TOF) MS and report the disease- and pregnancy-associated changes of 78 *N*-glycan species and 91 glycosylation traits derived thereof.

## Materials and Methods

### Study Population and Sample Collection

The current research is embedded in the PARA study, a nationwide prospective cohort on pregnancy and RA (21, 31). The cohort consisted of serum samples of 253 pregnancies from 219 RA patients, collected between 2002 and 2009 (31). In addition, 32 pregnancies of healthy Caucasian volunteers without adverse obstetric histories were included and followed from the first trimester of pregnancy. Of each patient, at least one sample was obtained during pregnancy and one postpartum, with a minimum of three samples per patient. Only completed pregnancies were included and all patients fulfilled the 1987 ACR criteria for RA. Disease activity was assessed using the disease activity score (DAS) in 28 joints, incorporating the swollen joint count, the tender joint count and the C-reactive protein (CRP) level [DAS28(3)-CRP]. The study was in compliance with the Helsinki Declaration and was approved by the Ethics Review Board at the Erasmus University Medical Center, Rotterdam, the Netherlands. Written and informed consent was obtained from all research participants included in this study. For quality control purposes, 111 plasma standards (Visucon-F frozen normal control plasma, pooled from 20 human donors, citrated, and buffered with 0.02 M HEPES, obtained from Affinity Biologicals, Ancaster, ON, Canada) and 40 PBS blanks were distributed across the 21 96-well sample plates.

### Chemicals and Enzymes

SDS and analytical grade ethanol were obtained from Merck (Darmstadt, Germany). Disodium hydrogen phosphate dihydrate (Na_2_HPO_4_ × 2H_2_O), potassium dihydrogenphosphate (KH_2_PO_4_), sodium chloride, Nonidet P-40 substitute (NP-40), 1-hydroxybenzotriazole monohydrate 97% (HOBt), 50% sodium hydroxide (NaOH), and super-DHB were obtained from Sigma-Aldrich (Steinheim, Germany). HPLC-grade acetonitrile (ACN) was purchased from Biosolve (Valkenswaard, the Netherlands), and 1-ethyl-3-(3-(dimethylamino)propyl)carbodiimide (EDC) hydrochloride was obtained from Fluorochem (Hadfield, UK). Peptide-*N*-glycosidase F (PNGase F) was obtained from Roche Diagnostics (Mannheim, Germany) and ultrapure water (MQ) was generated from a Purelab Ultra system (Veolia Water Technologies, Ede, the Netherlands), which was maintained at 18.2 MΩ at 25°C.

### Enzymatic *N*-Glycan Release

*N*-Glycans were released from serum proteins using PNGase F, as was previously described (32). In summary, 6 µL serum of each sample was denatured by addition of 12 µL of 2% SDS, followed by incubation for 10 min at 60°C. The release step was performed by the addition of 12.6 µL releasing mixture, which consisted of 2.5× PBS containing 2% NP-40 and 0.4 mU PNGase F, followed by 16 h incubation at 37°C. After release, the samples were stored at −20°C or transferred to the robot platform to be used directly.

### Glycan Derivatization and Purification

Further sample preparation was performed on an automated system, as previously described (30). In short, linkage-specific sialic acid stabilization (ethyl esterification) was performed by adding 60 µL 250 mM EDC 250 mM HOBt in ethanol to 3 µL released glycan sample. This mixture was incubated for 75 min at room temperature, after which 120 µL ACN was added. To purify the samples, a GHP membrane (GHP plate, Pall AcroPrep Advance 96 Filter plate, Pall Corporation, Ann Arbor, MI, USA) was prewetted with 70% ethanol, activated with MQ and equilibrated with 100% ACN. The complete sample was transferred to the GHP plate and incubated for 5 min. Subsequently the GHP plate was washed three times with 100 µL 96% ACN. Elution was performed by adding 30 µL of MQ to the GHP plate, incubating at room temperature for 5 min, followed by centrifugation into a PCR plate.

### Matrix-Assisted Laser Desorption/Ionization Time-of-Flight Mass Spectrometry (MALDI-TOF-MS) Analysis

From each purified sample, 10 µL was premixed with 10 µL matrix consisting of 5 mg/mL super-DHB in 99% ACN with 1 mM NaOH. Of this mixture, 2 µL was spotted onto a MALDI target plate (800/384 MTP AnchorChip, Bruker Daltonics, Bremen, Germany) and was left to dry. MALDI-TOF-MS spectra were recorded on an UltrafleXtreme mass spectrometer with a Smartbeam-II laser (Bruker Daltonics) in reflectron positive mode, controlled by flexControl 3.4 (Build 135). Measurements were performed within a range from *m*/*z* 1,000 to 5,000, accumulating 10,000 laser shots at a frequency of 1,000 Hz and with 100 shots per raster spot using a random walking pattern.

### Data Preprocessing

Average spectra were separately created for all healthy samples and for all RA samples. Within these two averages, 98 signals were manually assigned to putatively originate from glycan compositions. Using flexAnalysis 3.4 (Bruker Daltonics), the spectra were transformed into text format (*x*, *y*). Massytools (version 0.1.7.1 beta) was used to perform calibration and integration of the text files (33). Specifically, calibration was performed with a high precision calibration list, requiring at least 5 calibrants to be present at a RMS *S*/*N* ≥ 6 (119 spectra were excluded during this step, which included all 40 blanks). The putative glycan structures were extracted covering 95% of the isotopic envelope, summing per isotope the background-corrected area.

Spectra with quality control values below 3 SD of the mean values [i.e., fraction of spectrum in analytes, fraction of analyte area above *S*/*N* 9, and highest main peak (H5N4E2) *S*/*N*] were excluded from further analysis (13 spectra). Putative glycan signals were excluded from further analysis if failing to be present in 15% or more spectra of any analytical group (healthy, RA, standard) with an *S*/*N* ≥ 6 and a ppm error of at most 15 (which led to the exclusion of 21 signals). After curation, we retained 1,841 spectra with 78 analytes (Table S1 in Supplementary Material). Single *N*-glycan areas were normalized to the sum of all areas. To prevent outlier influence on the data analysis, individual glycosylation values surpassing 5 SD from their mean were removed from further analysis (118 values). Derived traits were calculated from the single glycans (Table S2 in Supplementary Material).

### Data Analysis

Measurement repeatability was assessed by calculating the mean, SD, and CV for all glycans and derived traits within the plasma controls (Table S3 and Figure S1 in Supplementary Material). Statistical analyses were performed using R 3.1.2 in an environment of RStudio 0.98.1091 (RStudio Team, Boston, MA, USA) (34). Figures were annotated with glycan symbols created in GlycoWorkbench 2.1 following the nomenclature proposed by the Consortium for Functional Glycomics (35, 36). For all statistical analyses, glycosylation values were scaled and centered, making regression effect sizes (*B*) representative of a 1 SD change in the glycan value.

Association between glycosylation and, respectively, pregnancy, RA, and DAS28(3)-CRP was explored by mixed linear regression. To establish the effect of pregnancy on glycosylation, a binary time point classification was constructed between (A) the first and third trimesters of pregnancy (coded, respectively, as 0 and 1), (B) the third trimester of pregnancy and 6 weeks postpartum (respectively, 0 and 1), and (C) the third trimester of pregnancy and 26+ weeks postpartum (respectively, 0 and 1). With an added random intercept per individual, glycan variables were used as outcome variable and time point classification as predictor variable (model: glycosylation ~ β_1_·time point) (Table S4 in Supplementary Material). The effect of RA (healthy = 0; RA = 1) on glycosylation was modeled by applying a random intercept for all time points, using glycosylation as outcome variable and RA as predictor variable (model: glycosylation ~ β_1_·RA) (Table S5 in Supplementary Material). The effect of DAS28(3)-CRP on glycosylation was modeled by either having a random intercept per time point (to analyze between individuals) or a random intercept per individual (to analyze within individuals), using glycosylation as outcome variable and DAS28(3)-CRP as predictor variable [model: glycosylation ~ β_1_·DAS28(3)-CRP] (Table S6 in Supplementary Material).

To validate associations between glycosylation and disease activity during pregnancy, the difference in DAS28(3)-CRP [ΔDAS28(3)-CRP] and glycosylation (Δglycosylation) was established between the first trimester and third trimester of pregnancy (third trimester − first trimester). The association between these was established by linear regression [Δglycosylation ~ β_1_·ΔDAS28(3)-CRP] (Table S7 in Supplementary Material). This process was similarly performed for the determination of the relationship between ΔDAS28(3)-CRP and Δglycosylation during the postpartum period, which was established between the third trimester of pregnancy and 12 weeks postpartum time point (12 weeks postpartum − third trimester). The time point at 12 weeks postpartum was chosen for the calculation because it displayed the highest disease activity after delivery, since most patients had already restarted anti-rheumatic therapy afterward.

Throughout the study, the Benjamini–Hochberg procedure was employed to control for multiple testing, which, under a study-wide false discovery rate of 5%, resulted in a significance threshold of α = 3.03 × 10^−2^ (Table S8 in Supplementary Material) (37).

## Results

**Figure 2:**
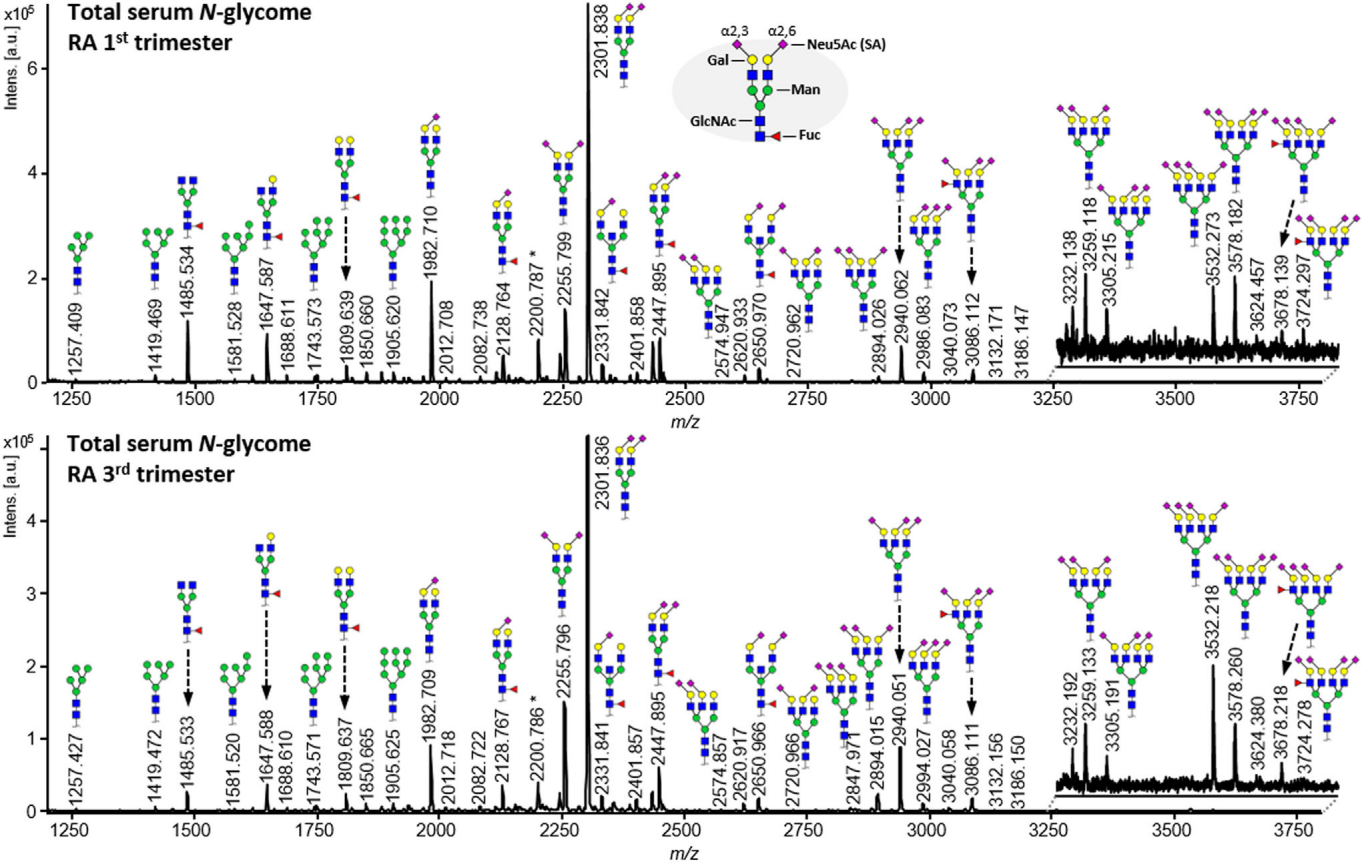
Comparison of matrix-assisted laser desorption/ionization time-of-flight mass spectrometry spectra obtained from released *N*-glycans from serum proteins after linkage-specific sialic acid esterification and HILIC enrichment. The shown spectra are derived from an individual with rheumatoid arthritis who has been diagnosed with ACPA and RF, and has been classified as a responder by the EULAR response criteria. Notable differences between the first trimester (*top*) and third trimester (*bottom*) spectra include galactosylation (e.g., between *m*/*z* 1,485.5, 1,647.6, and 1,809.6), antennarity (e.g., *m*/*z* 2,940.1 vs. 2,301.8), α2,3-linked sialylation (e.g., *m*/*z* 2,255.8 vs. 2,301.8), and α2,6-linked sialylation (e.g., *m*/*z* 1,982.7 vs. 2,301.8).

To explore the association between serum protein *N*-glycosylation and improvement of RA disease activity during pregnancy, we investigated by MALDI-TOF-MS the TSNG of 285 pregnancies embedded in the PARA study (characteristics of the study population can be found in Table 1, medication details in Table 2) (31). This MS methodology allowed us to obtain information on 78 glycan compositions (Figure 2; Table S1 in Supplementary Material), including discrimination between sialic acid linkage isomers, and to calculate biologically relevant ratios between subsets of glycans in the form of 91 derived traits (Table S2 in Supplementary Material) (29). Data quality was confirmed by the repeated measurement of a standard sample (Figure S1 and Table S3 in Supplementary Material).

**Table 1:** Study population characteristics.

	Control pregnancies (*n* = 32)	Patient pregnancies (*n* = 253)
Age at delivery in years, mean (SD)	32.1 (4.4)	32.8 (3.7)
Duration of pregnancy in weeks, mean (SD)	40.1 (1.4)	39.2 (1.9)
Disease duration at first visit in years, mean (SD)	–	7.7 (6.3)
ACPA-positive patients, *n* (%)	–	153/253 (60.5)
RF-positive patients, *n* (%)	–	161/240 (67.1)
Erosive disease, *n* (%)	–	149/229 (65.1)
Response during pregnancy, *n* (%)	–	72/137 (52.6)
Flare during postpartum period, *n* (%)	–	72/232 (31.0)
Disease activity score [DAS28(3)-CRP] at first trimester of pregnancy, mean (SD)	–	3.6 (1.1)

**Table 2:** Medication.

	pc (*n* = 131)	tm1 (*n* = 225)	tm2 (*n* = 235)	tm3 (*n* = 240)	pp1 (*n* = 243)	pp2 (*n* = 243)	pp3 (*n* = 245)
Prednisone, *n* (%)	38/111 (34.2)	80/217 (36.9)	87/234 (37.2)	81/238 (34.0)	85/236 (36.0)	88/239 (36.8)	79/240 (32.9)
Sulfasalazine, *n* (%)	44/130 (33.8)	63/224 (28.1)	65/235 (27.7)	61/239 (25.5)	61/241 (25.3)	73/243 (30.0)	71/243 (29.2)
Hydroxychloroquine, *n* (%)	9/130 (6.9)	5/224 (2.2)	5/235 (2.1)	4/239 (1.7)	9/241 (3.7)	18/243 (7.4)	17/243 (7.0)
Methotrexate, *n* (%)	0/130 (0.0)	0/224 (0.0)	0/235 (0.0)	0/239 (0.0)	34/241 (14.1)	59/243 (24.3)	74/243 (30.5)
TNF-inhibitors, *n* (%)	5/130 (3.8)	0/224 (0.0)	0/235 (0.0)	0/239 (0.0)	13/241 (5.4)	23/243 (9.5)	29/243 (11.9)

*pc, preconception; tm1, first trimester; tm2, second trimester; tm3, third trimester; pp1, 6 weeks postpartum; pp2, 12 weeks postpartum; pp3, 26 weeks postpartum*.

### Pregnancy-Associated Glycosylation Changes

The association of pregnancy and the postpartum period with serum protein *N*-glycosylation was established by mixed linear regression. Within individuals, comparison was made between the first and third trimester (representative of pregnancy; respectively, coded 0 and 1; adjusted for individual), third trimester and 6 weeks postpartum, and third trimester and 26+ weeks postpartum (Table S4 in Supplementary Material).

**Figure 3:**
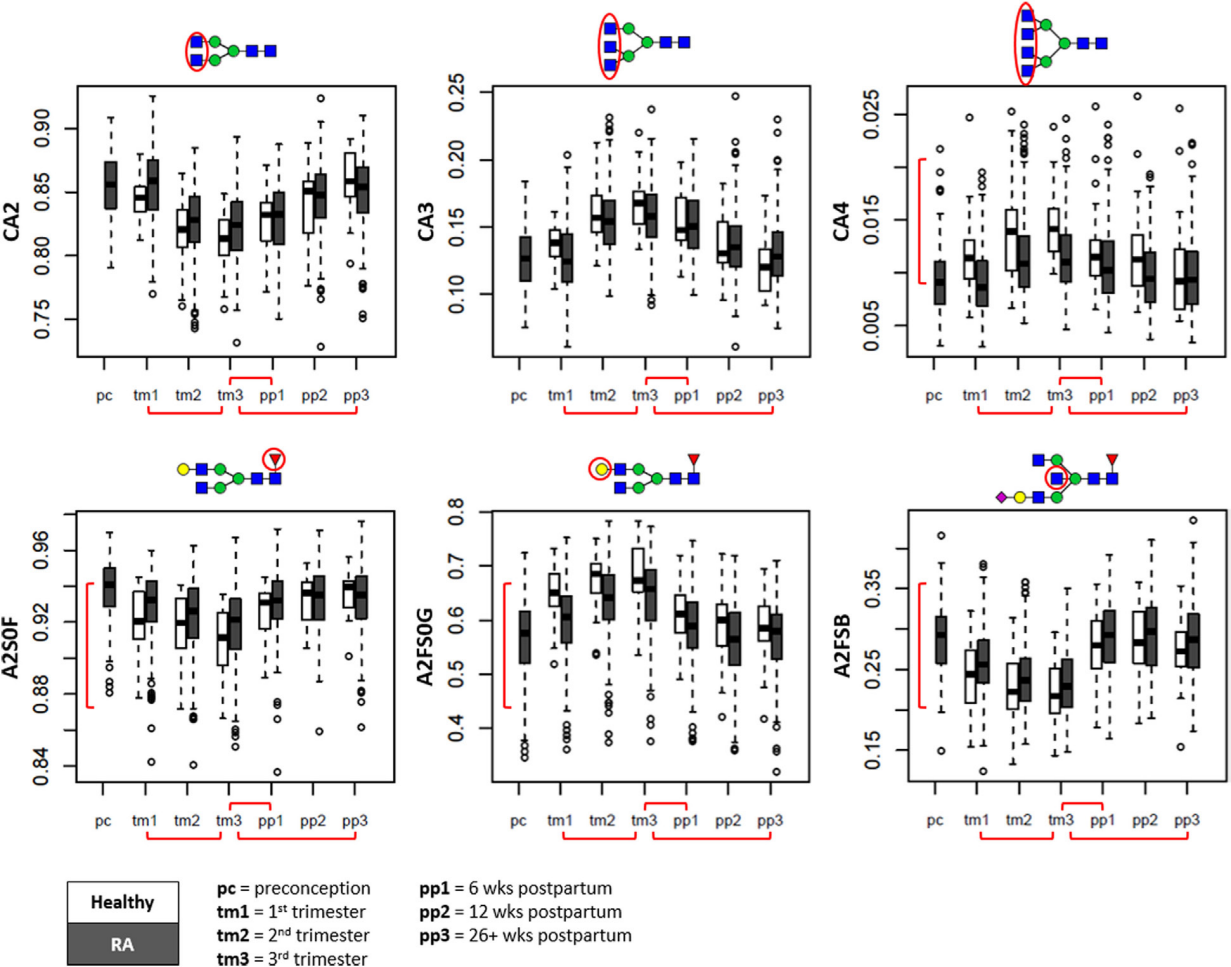
Overall glycosylation changes throughout pregnancy and the postpartum period shown in boxplots. Displayed are the glycan traits related to antennarity (*top row*), and the glycosylation traits likely to be of immunoglobulin origin (*bottom row*) (15, 26). Depicted are healthy controls (in white) and patients with rheumatoid arthritis (RA) (in gray) at preconception (pc), trimesters 1 through 3 (tm1, tm2, tm3), 6 weeks postpartum (pp1), 12 weeks postpartum (pp2), and 26+ weeks postpartum (pp3). CA2, diantennary species within complex type; CA3, triantennary species within complex type; CA4, tetraantennary species within complex type; A2S0F, fucosylation of non-sialylated diantennary species; A2FS0G, galactosylation of non-sialylated fucosylated diantennary species; A2FSB, bisection of sialylated fucosylated diantennary species. Red brackets indicate statistically significant findings between time points (horizontal), or between healthy individuals and RA patients (vertical).

With progression of pregnancy, a marked alteration in overall number of antennae per *N*-glycan was observed (Figure 3, top row). During pregnancy, within the complex type glycans (C), the relative abundance of tri- and tetraantennary *N*-glycans (CA3, CA4) showed to increase at the expense of diantennary species (CA2) (β_CA2_ = −1.05 SE ± 0.06; β_CA3_ = 1.08 ± 0.06; β_CA4_ = 0.70 ± 0.06), followed by a slow recovery after delivery (β_CA2_ = 0.94 ± 0.07; β_CA3_ = −0.98 ± 0.07; β_CA4_ = −0.56 ± 0.08; Table S4 in Supplementary Material).

For the serum *N*-glycosylation characteristics likely originating from the Fc portion of IgG (diantennary *N*-glycans without sialylation; A2S0) (15, 26), we confirmed with progressing pregnancy a decrease in fucosylation (A2S0F, β_A2S0F_ = −0.59 ± 0.05) and increase in galactosylation (A2FS0G, β_A2FS0G_ = 0.59 ± 0.04), both rapidly reversing after delivery (β_A2S0F_ = 0.67 ± 0.04; β_A2FS0G_ = −0.81 ± 0.04) (Figure 3, bottom row).

For the serum *N*-glycosylation characteristics likely originating for a large part from IgG-Fab, IgM, and IgA (fucosylated diantennary *N*-glycans with sialylation; A2FS) (15, 26), we observed a decrease in bisection with trimester progression (A2FSB, β_A2FSB_ = −0.56 ± 0.04) and a rapid return to pre-pregnancy levels at 6 weeks postpartum (β_A2FSB_ = 1.11 ± 0.03).

**Figure 4:**
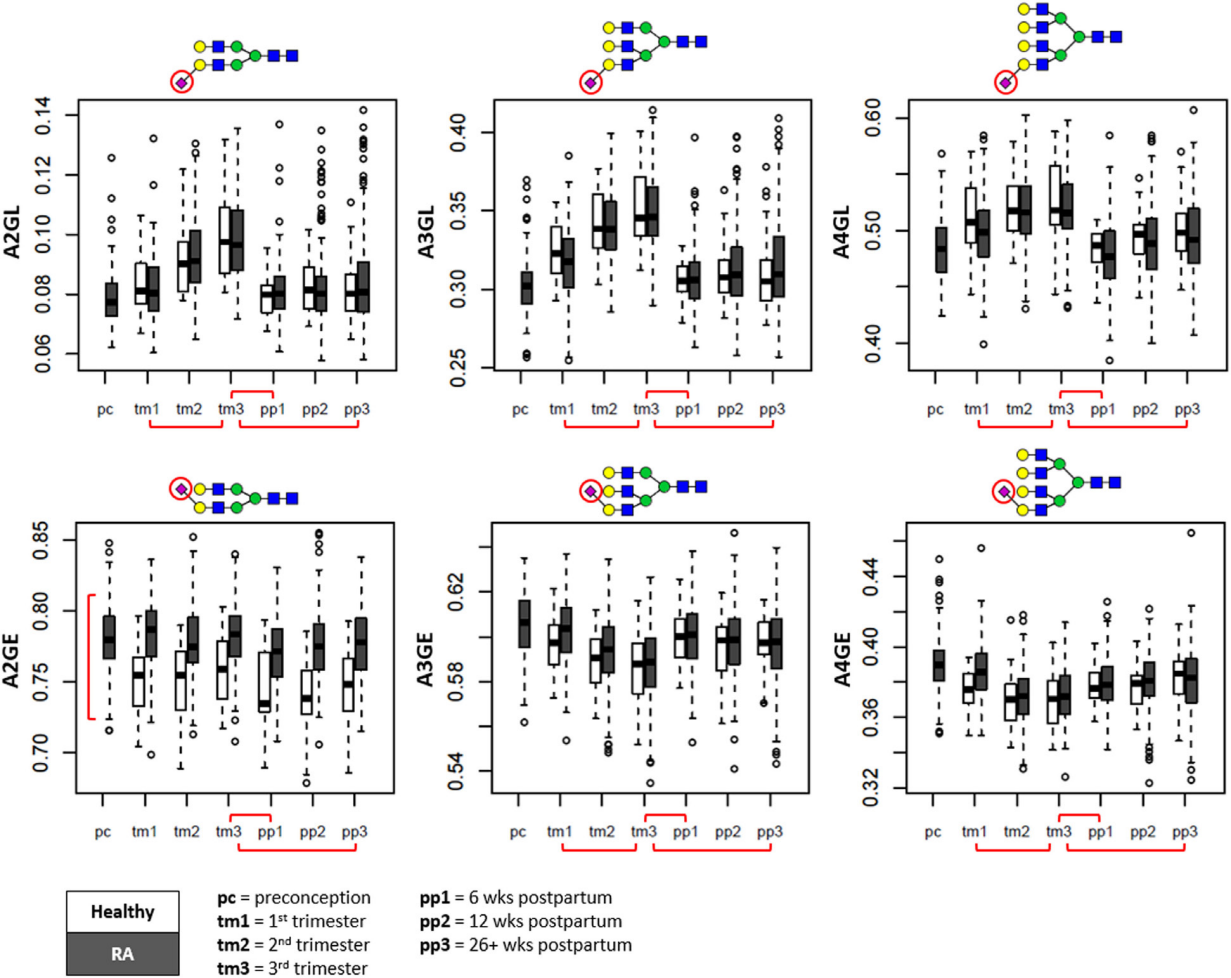
Sialylation changes throughout pregnancy and the postpartum period shown in boxplots. Whereas α2,3-linked sialylation (L) displays the most prominent change throughout pregnancy (*top row*), α2,6-linked sialylation shows the most distinction between patients and controls (*bottom row*). Separation is made between pregnancies of patients with rheumatoid arthritis (RA) (in gray) and healthy controls (in white). A2GL, α2,3-linked sialylation per galactose of diantennary species; A3GL, α2,3-linked sialylation per galactose of triantennary species; A4GL, α2,3-linked sialylation per galactose of tetraantennary species; A2GE, α2,6-linked sialylation per galactose of diantennary species; A3GE, α2,6-linked sialylation per galactose of triantennary species; A4GE, α2,6-linked sialylation per galactose of tetraantennary species. Red brackets indicate statistically significant findings between time points (horizontal), or between healthy individuals and RA patients (vertical).

Notably, whereas α2,6-linked sialylation remained relatively stable throughout pregnancy (Figure 4, bottom row), a drastic increase of α2,3-linked sialylation (e.g., per galactose on di- or triantennary glycans, respectively, A2GL and A3GL) was observed up to the third trimester (β_A2GL_ = 1.11 ± 0.05; β_A3GL_ = 1.16 ± 0.05), followed by a rapid return to baseline levels at the first time point after delivery (β_A2GL_ = −1.19 ± 0.05; β_A3GL_ = −1.42 ± 0.04) (Figure 4, top row).

### Differences between Healthy and RA

By mixed linear regression, glycosylation values were compared between individuals with RA and controls (healthy = 0, RA = 1; adjusted for pregnancy) (Table S5 in Supplementary Material). For immunoglobulin type glycosylation, we observed in RA a lower galactosylation (A2FS0G, β_A2FS0G_ = −0.44 ± 0.07), higher bisection (A2FS0B, β_A2FS0B_ = 0.55 ± 0.08), and a higher sialylation per galactose (A2FGS, β_A2FGS_ = 0.75 ± 0.08). In addition, differences were found in several non-fucosylated diantennary traits (higher A2F0G, A2F0GS, and A2GE, lower A2F0B, A2F), all mainly driven by elevated levels of H5N4E2 (β_H5N4E2_ = 0.97 ± 0.07) in RA patient serum. For tri- and tetraantennary *N*-glycosylation, we observe with RA a higher fucosylation (e.g., A3F, β_A3F_ = 0.34 ± 0.08; A4F, β_A4F_ = 0.56 ± 0.08) particularly within species with α2,3-linked sialylation (A3LF, β_A3LF_ = 0.35 ± 0.08; A4LF, β_A4LF_ = 0.58 ± 0.08), suggesting an increase in sialyl-Lewis X/A.

### Association of Glycosylation with DAS28(3)-CRP

In addition, we compared by mixed linear regression the association of glycosylation with RA disease activity, as assessed by DAS28(3)-CRP, both between and within individuals with RA (respectively, adjusted for time point and individual) (Figure S2 and Table S6 in Supplementary Material). The increase in DAS28(3)-CRP associated with both the decrease in IgG-Fc-type galactosylation (e.g., A2S0G, β_A2S0G_ = −0.34 ± 0.02) and the decrease in fucosylation of non-α2,3-sialylated triantennary species (A3L0F, β_A3L0F_ = −0.13 ± 0.03). Increasing with DAS28(3)-CRP were the bisection of IgG-Fc-type glycans (A2F0S0B, β_A2F0S0B_ = 0.25 ± 0.03) as well as the sialylation and fucosylation of tri- and tetraantennary species (e.g., A3FGS, β_A3FGS_ = 0.26 ± 0.02; A4F, β_A4F_ = 0.13 ± 0.03; A4GS, β_A4GS_ = 0.17 ± 0.02). Glycosylation features associating with DAS28(3)-CRP only within (and not between) individuals throughout pregnancy were the fucosylation of (α2,6-)sialylated diantennaries (A2SF, β_A2SF_ = −0.12 ± 0.02) and α2,6-sialylation of triantennary non-fucosylated species (A3F0GE, β_A3F0GE_ = 0.14 ± 0.02).

### Association of Glycosylation with the Improvement of DAS28(3)-CRP during Pregnancy

**Figure 5:**
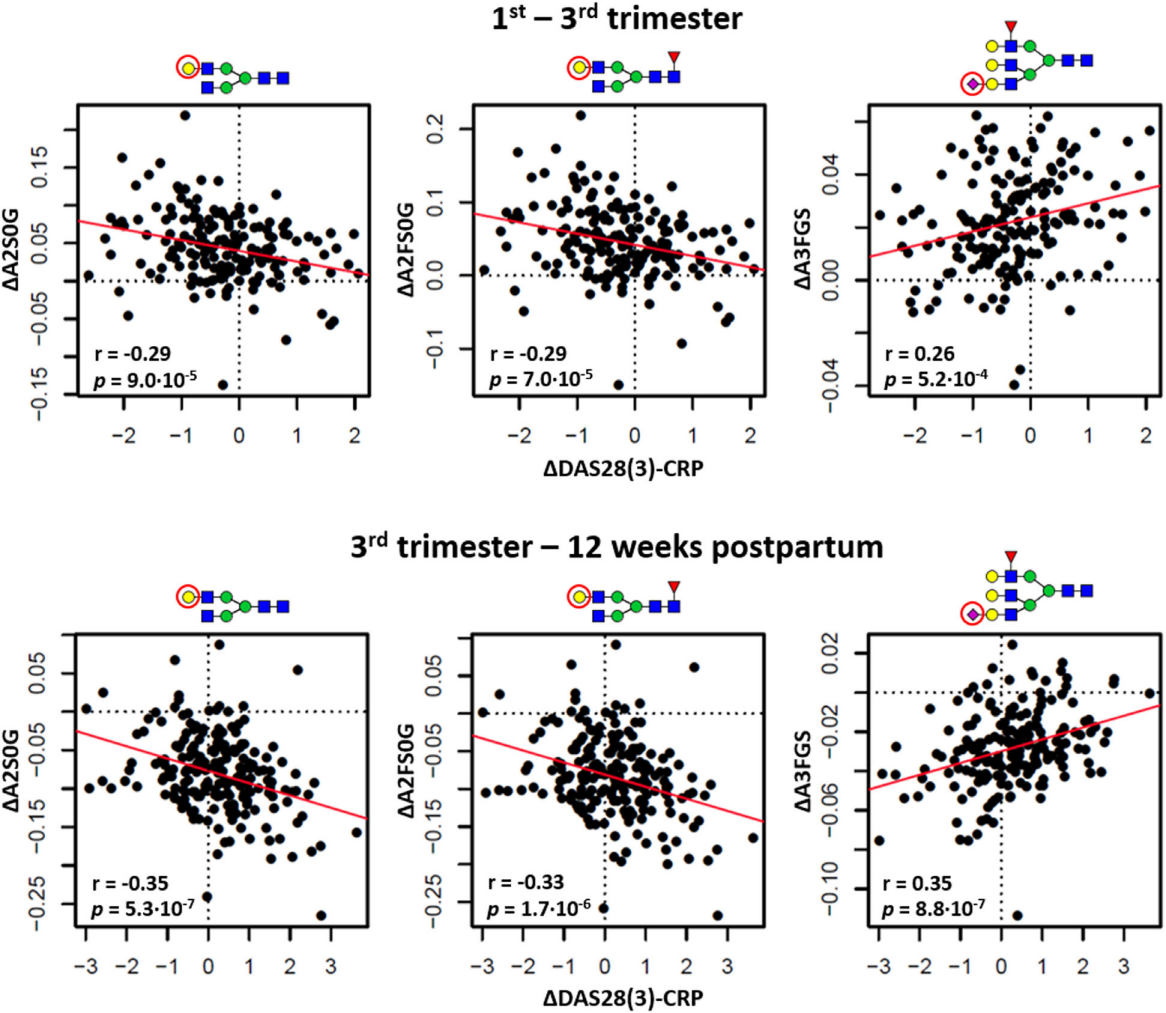
The relation between changing rheumatoid arthritis disease activity [ΔDAS28(3)-CRP] and changing glycosylation. Distinction is made between the timeframes to assess responder status (the change between first and third trimester; *top row*) and flare status (the change between third trimester and 12 weeks postpartum; *bottom row*). ΔA2S0G, the change in galactosylation per antenna of diantennary non-sialylated species; ΔA2FS0G, the change in galactosylation of diantennary fucosylated non-sialylated species; ΔA3FGS, the change in sialylation per galactose of triantennary fucosylated species. The significance of the models (*p*) arrives from linear regression, the correlation coefficients (*r*) from Pearson correlation.

Finally, between trimesters 1 and 3 (response timeframe) as well as between trimester 3 and 12 weeks postpartum (flare timeframe), we employed linear regression to confirm the within-individual association of DAS28(3)-CRP [ΔDAS28(3)-CRP] with glycosylation (Δglycosylation) (Table S7 in Supplementary Material). In both timeframes, ΔDAS28(3)-CRP showed a negative association with the Δgalactosylation of IgG-Fc type species (ΔA2S0G, responder, β_ΔA2S0G_ = −0.29 ± 0.07; flare, β_ΔA2S0G_ = −0.35 ± 0.07; ΔA2FS0G, responder, β_ΔA2FS0G_ = −0.29 ± 0.07; flare, ΔA2FS0G; β_ΔA2FS0G_ = −0.33 ± 0.07), whereas the sialylation of triantennary fucosylated species showed in both cases a positive association (ΔA3FGS, responder, β_ΔA3FGS_ = 0.26 ± 0.07; flare, β_ΔA3FGS_ = 0.35 ± 0.07) (Figure 5). The flare timeframe additionally showed a positive association of ΔDAS28(3)-CRP with the (α2,6)-sialylation of (fucosylated) diantennary glycans (ΔA2GE, β_ΔA2GE_ = 0.27 ± 0.07; ΔA2FGS, β_ΔA2FGS_ = 0.24 ± 0.07).

## Discussion

The decrease of RA disease activity during pregnancy and the flare following delivery are reproducible clinical observations that are mechanistically poorly understood (8–10). To expand our understanding of protein glycosylation that associates with RA disease activity, we studied the total serum *N*-glycosylation changes occurring throughout pregnancy and the postpartum period of 253 pregnancies of patients with RA, along with 32 control pregnancies.

### Interpretation of Glycomics Data

To interpret the information contained in this study, several aspects of our MALDI-TOF-MS analysis need to be kept in mind. First, MS assesses glycan chemical compositions and not structures, although sialic acid linkage information is provided by the employed derivatization technique (29). Other structural characteristics are presumed based on a wide array of literature on biosynthetic pathways, and on experiments with enzymatic digestion, nuclear magnetic resonance spectroscopy, and MS fragmentation (12, 15, 38–40). Second, the changes observed in the released *N*-glycan samples could have originated not only from changes in the glycosylation of proteins but also from changes in the abundance of those glycoproteins. Derived traits have been constructed to reflect differences in biosynthesis, and our current day understanding of the relative contribution of specific glycoproteins and tissues to the serum *N*-glycome (15, 26). Third, the mass spectrometric analysis did not provide quantitative ratios of *N*-glycosylation, but the direction and magnitude of observed changes is expected to be biologically representative, as suggested by method comparisons (41).

In addition, it has to be noted that the clinical associations found in the study may, in part, originate from treatment differences between individuals during the study period. For example, methotrexate treatment typically only starts after delivery, potentially influencing the time point comparisons. However, this confounding effect is expected to be limited due to the relatively low number of individuals undergoing the treatment. Details on the medication can be found in Table 2.

### Glycosylation Changes with Pregnancy

Previously, we found glycosylation changes throughout healthy pregnancies, and have in RA patients studied the specific glycosylation of IgG-Fc throughout pregnancy (20, 21, 27, 28, 42, 43). In the current study, the first application of a similar MALDI-MS approach with automated sample preparation, we achieved the analysis of the total serum *N*-glycosylation throughout the pregnancies of both healthy controls and RA patients, in total leading to the analysis of 1,770 clinical samples (30). Since the current cohort also contains the control individuals (without RA) from prior studies, these previous results were confirmed, but we could also for the first time show that comparable TSNG changes can be observed in RA patients.

As such, we detected with the progression of pregnancy an increase of galactosylation (A2FS0G) as prior reported for the Fc part of IgG, as well as a decrease in bisection (A2FSB) (18, 20, 21). Additionally, we observed with pregnancy an overall increase in glycan branching (from CA2 to CA3 and CA4) and an increase of α2,3-linked sialylation (L, in part at the expense of α2,6-sialylation, E). In all cases, the postpartum period led to the return to the values before or at the beginning of pregnancy. The increased branching observed in the TSNG could have originated from the abundance and antennarity of acute-phase proteins such as alpha-1-acid glycoprotein, for which an increased serum level and *N*-glycan branching has been reported with pregnancy (24, 25). On the other hand, we did not observe the decrease in fucosylation reported for the same protein, potentially obscured by the increased fucosylation of other proteins, and have yet to identify the source of the substantial increase of α2,3-linked sialylation up to the third trimester. In literature, it has been reported that IgG and alpha-1-acid glycoprotein glycosylation may be affected by estrogens (44, 45), known to change significantly throughout pregnancy (46), and it is conceivable that other proteins within the TSNG are under similar high-level control.

### Glycosylation Differs between RA Patients and Healthy Controls

The total serum *N*-glycosylation changes with pregnancy showed remarkably comparable between RA patients and healthy controls, but baseline differences could be detected. For example, RA patients displayed a lower degree of IgG-Fc-type galactosylation and higher bisection when compared to controls (A2FS0G, A2FS0B). A decreased IgG-Fc galactosylation and increased bisection are well-known to associate with a variety of inflammatory conditions, including RA, inflammatory bowel disease, and aging (18, 47, 48), and the same glycosylation phenotypes appear detectable in our TSNG study as well (20, 21). The mechanisms by which IgG-Fc glycosylation may affect inflammatory processes remain for a large part to be elucidated, but increased galactosylation and decreased fucosylation have been implicated in increased FcγRIIa and FcγRIIIa binding and antibody-dependent cellular cytotoxicity (49–51).

Furthermore, RA patients showed in our study a higher α2,6-sialylation and lower fucosylation of diantennary glycans (A2GE, A2F) compared to healthy controls, mainly driven by the *N*-glycan composition H5N4E2, as well as a substantially higher (multi-)fucosylation of tri- and tetraantennary glycans [A3F(a), A4F(a)]. The increased A3/A4 fucosylation strongly suggests the upregulation of sialyl-Lewis X, which has been reported with inflammatory arthritis for several acute-phase proteins, e.g., alpha-1-acid glycoprotein, haptoglobin, alpha-1-antichymotrypsin, and transferrin (52–56). The sialyl-Lewis X on glycans is known to bind to E-selectin, an inducible receptor expressed by endothelial cells (16). This interaction is implicated in the homing of immune cells to a site of inflammation (57), as well as cancer metastasis (58), and may play a role in RA as well. For instance, the alpha-1-acid glycoprotein observed within RA synovial fluid is thought to be of hepatic origin (59), meaning its circulatory variant might make use of a glycan-mediated mechanism for transportation toward the inflamed synovial tissue.

### Glycosylation Associates with RA Disease Activity

Next to a negative association with the galactosylation of IgG-Fc-type *N*-glycans, we additionally report a positive association between (Δ)DAS28(3)-CRP and the sialylation of triantennary fucosylated glycans (A3FGS), notably of the α2,6-linked variety. This is in contrast to the TSNG changes throughout pregnancy, which shows marked increase of α2,3-linked sialylation but not an association with disease activity. A recent study, which compared total plasma *N*-glycosylation with the levels of various metabolic and inflammatory markers, has indicated a link between A3FGS and CRP, suggesting that the CRP component of DAS28(3)-CRP may in part be responsible for the association found within the current study (60). While the protein source of A3FGS remains unknown, future studies will have to reveal its biomarker potential and facilitate our understanding of RA disease severity.

### Summary

To summarize, by performing MS-based total serum protein *N*-glycosylation analysis we detected (1) changes in protein glycosylation throughout pregnancy, (2) glycosylation differences between healthy individuals and RA patients, and (3) glycosylation traits coinciding with the pregnancy-associated changes in RA disease activity. While we confirmed in serum the IgG glycosylation phenotypes that were prior reported to associate with RA disease activity, our glycomics approach has additionally allowed the detection of changes that are presumably independent from IgG, namely, the sialylation of fucosylated triantennary *N*-glycans (A3FGS).

## Ethics Statement

The study was in compliance with the Helsinki Declaration and was approved by the Ethics Review Board at the Erasmus University Medical Center, Rotterdam, the Netherlands. Written and informed consent was obtained from all research participants included in this study.

## Author Contributions

All authors contributed to writing the manuscript. KR, GV, and AB designed and performed the experiments and analyzed the data. MB and YB facilitated the automated sample preparation. JH and RD collected and maintained the clinical cohort. MW and RD supervised the study.

## Conflict of Interest Statement

KR and MW are inventors on a patent application on sialic acid derivatization by ethyl esterification. All other authors declare that the research was conducted in the absence of any commercial or financial relationships that could be construed as a potential conflict of interest.
