# Supplementary material for: Serum Protein N-Glycosylation Changes with Rheumatoid Arthritis Disease Activity during and after Pregnancy
Source: Front Med (Lausanne). 2018 Jan 8;4:241. doi: 10.3389/fmed.2017.00241 (PMC5766648; doi:10.3389/fmed.2017.00241)
Supplement: Supplementary file 2 [file Image_1.PDF]

Supplementary figures

Serum protein *N*-glycosylation changes with rheumatoid arthritis disease activity during and after pregnancy

Karli R. Reiding[1]*, Gerda C. M. Vreeker[1], Albert Bondt[1,2], Marco R. Bladergroen[1], Johanna M. W. Hazes[3], Yuri E. M. van der Burgt[1,4], Manfred Wuhrer[1], Radboud J. E. M. Dolhain[3]

[1]*Center for Proteomics and Metabolomics, Leiden University Medical Center, Leiden, The Netherlands;*

[2]*Department of Rheumatology, Leiden University Medical Center, Leiden, The Netherlands;*

[3]*Department of Rheumatology, Erasmus University Medical Center, Rotterdam, The Netherlands;*

[4]*Department of Clinical Chemistry, Leiden University Medical Center, Leiden, The Netherlands;*

*To whom correspondence should be addressed:

Karli R. Reiding; Email: k.r.reiding@lumc.nl; Tel.: +31-71-52-68701; P.O. Box 9600, 2300 RC Leiden, The Netherlands

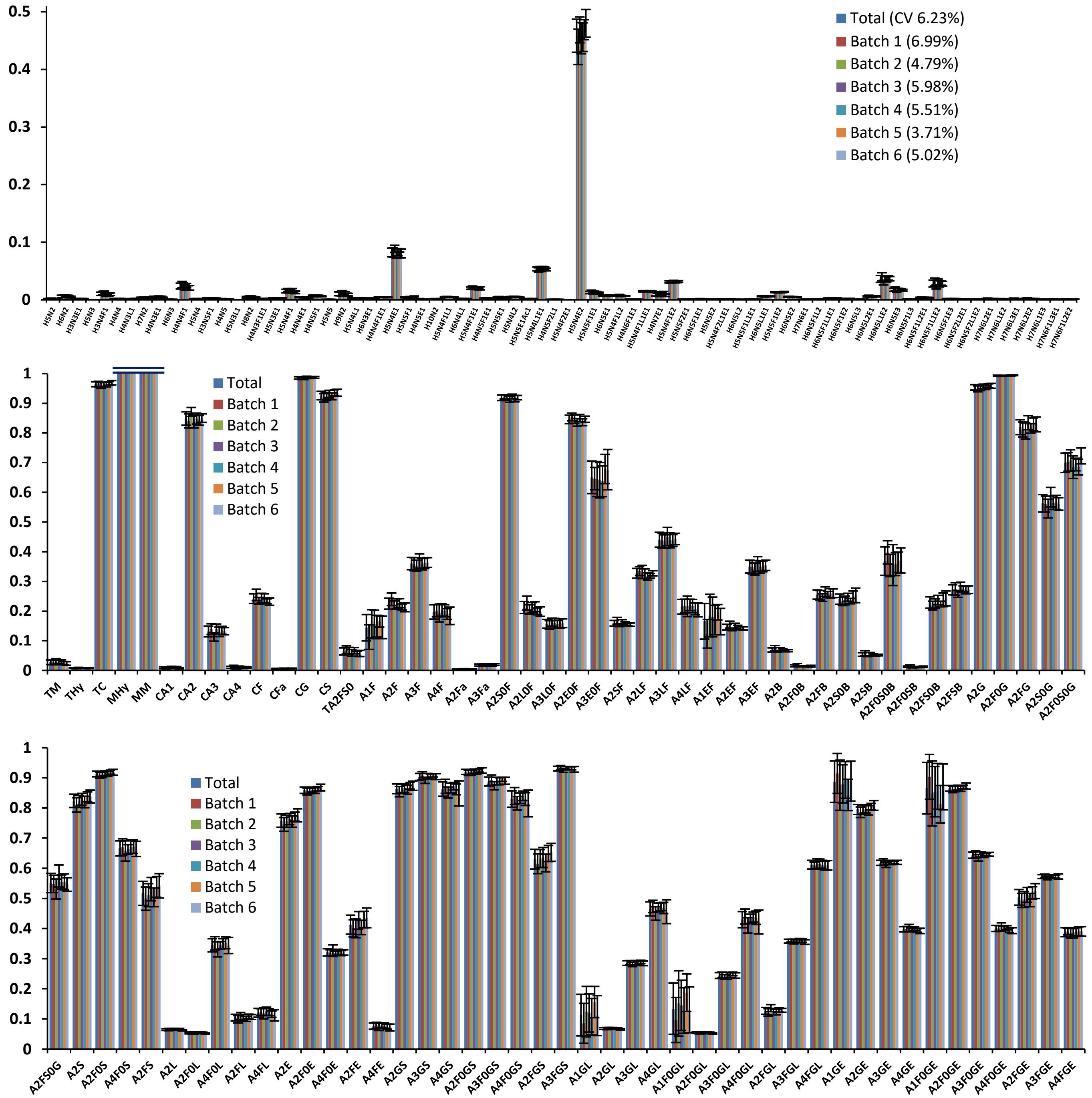

Figure S1. Overview of the variability and batch-to-batch variation of the mass spectrometric total serum *N*-glycomics analysis. Shown here are the mean values and standard deviation of a single technical replicate plasma sample distributed across the 6 measurement batches (MALDI-TOF-MS target plates) for single *N*-glycans species (**top**), and derived glycosylation traits (**middle** and **bottom**). The coefficient of variation has been established on the main peak (H5N4E2). In total the data shown here reflects the analysis of 111 repeat analysis of the same plasma standard.

Supplemental Figure S1

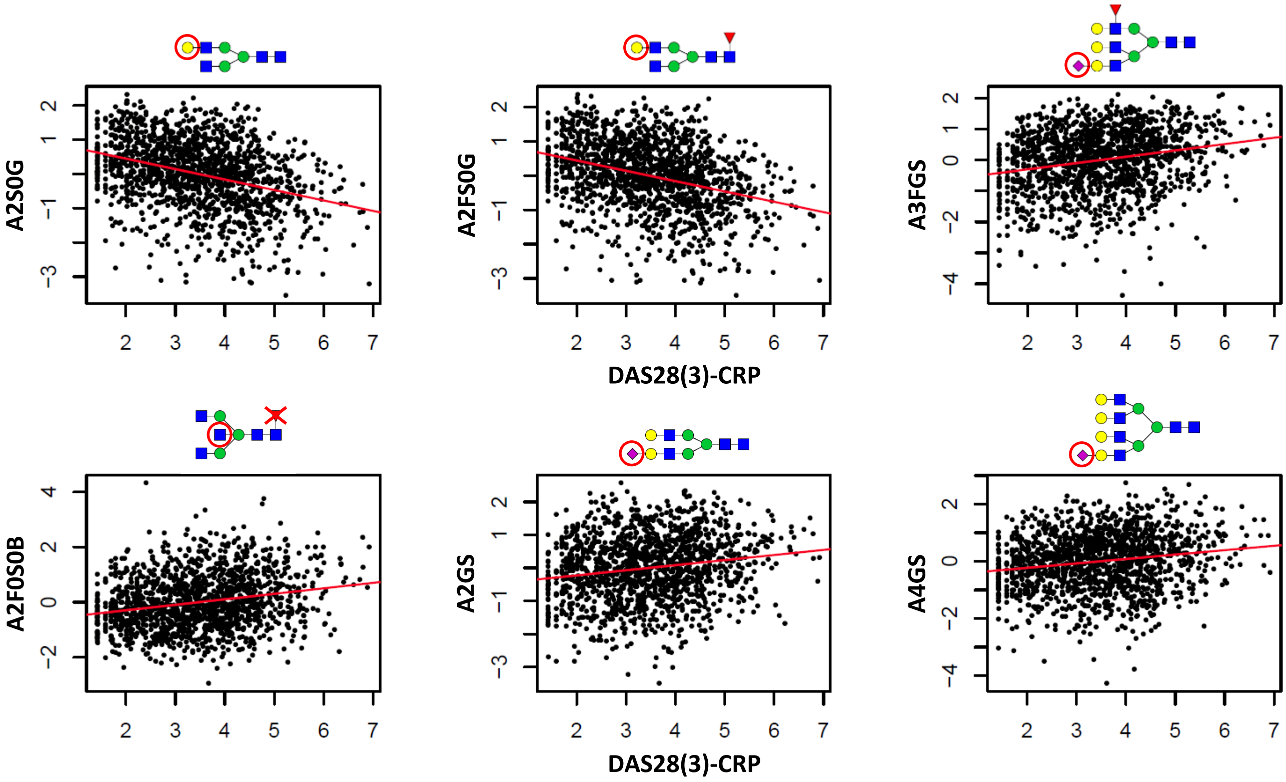

Figure S2. Association of glycosylation traits with RA disease activity (DAS28(3)-CRP). A2S0G = galactosylation per antenna of diantennary nonsialylated species; A2FS0G = galactosylation of diantennary fucosylated nonsialylated species; A3FGS = sialylation per galactose of triantennary fucosylated species; A2F0S0B = bisection of nonsialylated nonfucosylated diantennary species; A2GS = sialylation per galactose of diantennary species; A4GS = sialylation per galactose of tetraantennary species

Supplemental Figure S2
